# Derivation and validation of a prognostic model for predicting in-hospital mortality in patients admitted with COVID-19 in Wuhan, China: the PLANS (platelet lymphocyte age neutrophil sex) model

**DOI:** 10.1186/s12879-020-05688-y

**Published:** 2020-12-17

**Authors:** Jiong Li, Yuntao Chen, Shujing Chen, Sihua Wang, Dingyu Zhang, Junfeng Wang, Douwe Postmus, Hesong Zeng, Guoyou Qin, Yin Shen, Jinjun Jiang, Yongfu Yu

**Affiliations:** 1 MOE-Shanghai Key Laboratory of Children’s Environmental Health, Xin Hua Hospital Affiliated to Shanghai Jiao Tong University School of Medicine, Shanghai, China; 2 Department of Epidemiology, University Medical Center Groningen, Groningen, The Netherlands; 3 Department of Pulmonary and Critical Care Medicine, Zhongshan Hospital, Fudan University, Shanghai, China; 4 Department of Thoracic Surgery, Union Hospital, Tongji Medical College, Huazhong University of Science and Technology, Wuhan, China; 5 Department of Tuberculosis and Respiratory Disease, Jinyintan Hospital, Wuhan, China; 6 Julius Center for Health Science and Primary Care, University Medical Center Utrecht, Utrecht University, Utrecht, The Netherlands; 7 Department of Cardiology, Tongji Hospital, School of Medicine, Huazhong University of Science and Technology, Wuhan, China; 8 Department of Biostatistics, School of Public Health, and The Key Laboratory of Public Health Safety of Ministry of Education, Fudan University, Shanghai, China; 9 Eye Center, Medical Research Institute, Wuhan University Renmin Hospital, Wuhan University, Wuhan, China

**Keywords:** COVID-19, In-hospital mortality, Prognostic model, PLANS

## Abstract

**Background:**

Previous published prognostic models for COVID-19 patients have been suggested to be prone to bias due to unrepresentativeness of patient population, lack of external validation, inappropriate statistical analyses, or poor reporting. A high-quality and easy-to-use prognostic model to predict in-hospital mortality for COVID-19 patients could support physicians to make better clinical decisions.

**Methods:**

Fine-Gray models were used to derive a prognostic model to predict in-hospital mortality (treating discharged alive from hospital as the competing event) in COVID-19 patients using two retrospective cohorts (*n* = 1008) in Wuhan, China from January 1 to February 10, 2020. The proposed model was internally evaluated by bootstrap approach and externally evaluated in an external cohort (*n* = 1031).

**Results:**

The derivation cohort was a case-mix of mild-to-severe hospitalized COVID-19 patients (43.6% females, median age 55). The final model (PLANS), including five predictor variables of platelet count, lymphocyte count, age, neutrophil count, and sex, had an excellent predictive performance (optimism-adjusted C-index: 0.85, 95% CI: 0.83 to 0.87; averaged calibration slope: 0.95, 95% CI: 0.82 to 1.08). Internal validation showed little overfitting. External validation using an independent cohort (47.8% female, median age 63) demonstrated excellent predictive performance (C-index: 0.87, 95% CI: 0.85 to 0.89; calibration slope: 1.02, 95% CI: 0.92 to 1.12). The averaged predicted cumulative incidence curves were close to the observed cumulative incidence curves in patients with different risk profiles.

**Conclusions:**

The PLANS model based on five routinely collected predictors would assist clinicians in better triaging patients and allocating healthcare resources to reduce COVID-19 fatality.

## Background

The novel coronavirus disease 2019 (COVID-19) has become a pandemic worldwide since its first outbreak in Wuhan, China since December 2019 [1]. As of July 3, 2020, more than 10 million cases are confirmed in over 200 countries, including 517,337 deaths [2]. Due to the high contagiousness and rapid progression of the disease, healthcare demand, in particular for critical care capacities, has often been overwhelming even in high-income areas [3]. Good support tools are needed for clinicians and other healthcare workers to respond promptly to urgent situations. It is crucial to accurately select severe patients for targeted treatment. For example, while it is essential to increase the intensive care unit (ICU) capacities and staff, ICU triage may be critical to prioritize severe patients for intensive care [4]. Therefore, early stratification of patients will facilitate targeted supportive care and appropriate allocation of medical resources.

Prognostic model that combines several clinical or non-clinical variables to estimate the future health outcomes of an individual could be a useful tool [5]. To respond quickly to the health crisis of COVID-19, a prognostic model based on robust evidence could be used as a simple and inexpensive tool to assist physicians in triaging the patients in the first place, which in turn may mitigate the burden of overwhelmed healthcare system and better allocate limited healthcare resources to reduce COVID-19 fatality [6]. Currently, several clinical prognostic models have been developed for COVID-19 patients [7, 8]. However, the quality of these models has been criticized and was prone to bias due to unrepresentativeness of patient population, lack of external validation, inappropriate statistical analyses, or poor reporting [7]. Two of these prognostic models have been constructed with promising predictive performance for predicting mortality [9, 10]. However, they may not be highly reliable due to relatively small derivation cohorts (189 to 296 patients) and external validation cohorts (19 to 165 patients). Several studies used a time-to-event analysis to allow for administrative censoring [11–13]. However, censoring for other reasons, such as being discharged alive because of quick recovery, were seldom considered to be analyzed in a competing risk framework. For example, the CALL score [11] predicted the disease progression in hospitalized COVID-19 patients by using the standard Cox model. In this study, the risk of progression would be over-estimated because the patients discharged alive are no longer at risk of disease progression while the standard Cox model assumes they are still at risk.

In this study, we aimed to develop and validate a prognostic model to predict in-hospital mortality in COVID-19 patients using routinely measured demographic and clinical characteristics.

## Methods

### Study cohorts

#### Derivation cohort

The derivation cohort included 1008 COVID-19 patients admitted at Jinyintan Hospital (*n* = 763) and Union Hospital (*n* = 245) in Wuhan, China from January 1 to February 10, 2020. Patients were followed up to March 20, 2020. Patients who were still hospitalized until March 20, 2020 were not included in the analyses. The Jinyintan hospital had mostly severe patients while Union Hospital had mostly mild patients, thus providing a case-mix of mild-severe COVID-19 patients.

#### Validation cohort

The validation cohort included 1031 COVID-19 patients aged ≥18 years at Tongji Hospital in Wuhan, China from January 14 to March 8, 2020. Since this cohort was designed to assess the potential risk factors related to acute cardiac injury in COVID-19 patients, patients with stage of chronic kidney disease ≥4, chronic heart failure in the decompensatory stage, acute myocardial infarction during hospitalization, or having missing information on hypersensitive cardiac troponin I were excluded. Patients were followed up to March 30, 2020.

### Data collection

A trained team of physicians retrospectively reviewed clinical electronic medical records and laboratory findings for all the patients. All patients met the diagnostic criteria according to the WHO interim guidance [14]. In the derivation cohort, we collected data on age, sex, the dates of admission and discharge or death, complete blood count at admission (neutrophil, lymphocyte, platelet count, haemoglobin), current smoking status (no, yes), chronic disease history (hypertension, digestive disease, kidney disease, coronary heart disease (CHD), chronic pulmonary disease, cerebrovascular disease, diabetes, thyroid disease, malignancy, and other diseases). In the validation cohort, we collected data on age, sex, the dates of admission and discharge or death, complete blood count at admission (neutrophil, lymphocyte, platelet count), chronic disease history (hypertension, diabetes, CHD). All data were reviewed and collected by two physicians and a third researcher adjudicated any difference in interpretation between the two physicians.

### Outcome and candidate predictors

The end point of interest was the time from hospital admission until in-hospital death (event of interest) or discharged alive (competing event) or 30-day after hospital admission (censored), whichever came first. Discharged alive was treated as a competing event because the event of discharged alive precludes the event of in-hospital death. Since conventional survival methods, such as Kaplan-Meier method and Cox model, assume two competing events (in-hospital death and discharged alive) are independent, they are not valid any more and more advanced methods accounting for competing risks should be used. Candidate predictor variables included a set of demographic variables (age, sex, current smoking status), laboratory findings (neutrophil count, lymphocyte count, platelet count), and comorbidities (hypertension, CHD, diabetes, cerebrovascular disease, and malignancy), which were selected according to clinical knowledge, literature, [7, 15] and data availability. While current smoking status was not considered due to high proportion of missing data in the derivation cohort (46.3% missing), information on all other candidate predictor variables and outcome was complete for data analysis.

### Model derivation

Fine-Gray models were used to develop the prognostic model, treating discharged alive from hospital as a competing event [16]. The prognostic model derivation consisted of a prognostic index (PI) that captured the effect of the predictor variables on cumulative incidence function (CIF) for death, and a baseline CIF that determined the cumulative mortality of an “average” patient, i.e., a patient with the average value of PI. First, uni-variable Fine-Gray models with fractional polynomials (maximum permissible degree 1) were performed to investigate the potential non-linear relationship between continuous variables and CIF for death. Second, a multivariable Fine-Gray model with all the predictors was built. Backward elimination was applied to do the variable selection with significant level setting to 0.05, resulting in a final model in this step. PI was then calculated based on the combination of β coefficients and values of the corresponding predictors. The baseline CIF CIF_0_(*t*) corresponds to the cumulative mortality of an “average” patient with the average value of PI. The CIF for death of other patients can be computed via the formula: ${\mathrm{CIF}}_i(t)=1-{\left(1-{\mathrm{CIF}}_0(t)\right)}^{\exp \left({PI}_i-\overline{PI}\right)}$, where *PI*_*i*_ is the PI of patient i and $\overline{PI}$ is the average value of PI in the derivation cohort. Details about the implementation and estimates of the Fine-Gray model, see the Additional file 1: Appendix Text 1.

### Model performance and internal validation

Model performance was assessed in terms of discrimination and calibration. Discrimination was assessed using the concordance statistic (C-index) [17]. Calibration was assessed jointly by calibration slope and calibration plot. Calibration slope is a measure to estimate the regression coefficient on the PI in the validation dataset [18]. In the calibration plot, the averaged predicted mortality curves estimated by the proposed prognostic model were compared with the averaged observed mortality curves across several risk groups. The risk group was based on patients’ PI (thresholds: 16th, 50th and 84th percentiles) [19].

We performed internal validation to estimate the optimism (the level of model overfitting) and adjusted measures of C-index and calibration slope by bootstrapping 1000 samples of the original data (a detailed description of implementation of bootstrap is provided in Additional file 1: Appendix Text 2). Average calibration slope in the internal validation was obtained to be a uniform shrinkage factor. We multiplied the shrinkage factor by the raw PI (PI in the model derivation step) to obtain optimism-adjusted PI. Lastly, we developed the final model by re-estimating the baseline CIF for death based on the optimism-adjusted PI.

### External validation

The final model was applied to each patient in the external validation cohort. PI was then calculated based on the combination of β coefficients and the corresponding predictor values of every patient. The discriminative accuracy of the proposed model was evaluated using C-index and visually checked by the distribution of PIs. The calibration accuracy of the proposed model was assessed using calibration slope and visually checked by calibration plot.

### Statements about reporting and evaluation of our prognostic model

The reporting of this prognostic model study followed Transparent Reporting of a multivariable prediction model for Individual Prognosis Or Diagnosis (TRIPOD) statement (Additional file 2) [20]. The risk of bias of the prognostic model was independently assessed by an expert (JW, who did not take part in the model development and validation) using PROBAST (prediction model risk of bias assessment tool) [21].

## Results

### Patient population

In the derivation cohort, the median age of 1008 patients was 55 (interquartile range [IQR] 44–65, youngest at 14 years of age and oldest at 98 years) and 43.6% patients were females. During a median length of stay (LOS) of 12 days (IQR 8–16), 211 patients died in total, and 4 of which died beyond 30 days. Seven hundred fifty-seven patients discharged alive from the hospital within 30 days. There were 438 (43.5%) patients with one or more comorbidities. Hypertension (*N* = 232, 23.0%), diabetes (*N* = 110, 10.9%), chronic digestive disease (*N* = 78, 7.7%), and chronic pulmonary disease (*N* = 40, 4.0%) were among the most frequent comorbidities (Table 1).

**Table 1:** Basic characteristics

	Derivation cohort (*n* = 1008)	Validation cohort (*n* = 1031)
Age, years	55 (44–65)	63 (52–70)
Sex, female	439 (43.6%)	493 (47.8%)
Current smoke status^*^	57 (10.5%)	**–**
Neutrophil count, × 10^9^/L	4.40 (2.79–6.96)	3.90 (2.78–5.68)
Lymphocyte count^*^, × 10^9^/L	0.95 (0.61–1.34)	1.07 (0.70–1.49)
Platelet count^*^, × 10^9^/L	194 (145–256)	219 (164–288)
Haemoglobin^*^, g/L	126 (115–138)	**–**
Chronic pulmonary disease	40 (4.0%)	**–**
Hypertension	232 (23.0%)	383 (37.1%)
Coronary heart disease	32 (3.2%)	83 (8.1%)
Diabetes	110 (10.9%)	189 (18.3%)
Thyroid disease	31 (3.1%)	**–**
Chronic digestive disease	78 (7.7%)	**–**
Cerebrovascular disease	22 (2.2%)	**–**
Chronic kidney disease	25 (2.5%)	**–**
Malignancy	31 (3.1%)	**–**

^*^Current smoke status was missing in 467 (46.3%) patients in the derivation cohort, lymphocyte count was missing in 1 patient in the validation cohort, platelet count was missing in 2 patients in the validation cohort, and haemoglobin was missing in 376 (37.3%) patients in the derivation cohort

In the validation cohort, the 1031 patients included were older (63, IQR 52–70), had more females (47.8%), and were more prevalent with hypertension (*N* = 383, 37.1%), CHD (*N* = 83, 8.1%) and diabetes (*N* = 189, 18.3%), compared to the derivation cohort (Table 1). Patients had a longer LOS (19, IQR 11–27). The in-hospital mortality of patients in the validation cohort was slightly lower compared with the derivation cohort (Additional file 1: Appendix Fig. 1).

### Coding of predictors

Categorical predictors (sex, hypertension, CHD, diabetes, cerebrovascular disease and malignancy) were coded as dummy variables. Among continuous predictors, we did not observe obvious violation of linearity assumption for age, neutrophil and platelet count. We observed a non-linear relation between outcome and lymphocyte count. Therefore, we included the transformed lymphocyte count (square root of the lymphocyte count) in the model according to the results of fractional polynomial analyses.

### Model derivation and internal validation

The PLANS model included five predictors: platelet count, lymphocyte count, age, neutrophil count, and sex. Cumulative incidence function for the in-hospital mortality was associated with older age, being male, higher neutrophil, lower lymphocyte and lower platelet count (Table 2). This model showed excellent apparent discriminative ability (C-index: 0.85, 95% CI: 0.83 to 0.88). After adjusting for overfitting, the model maintained excellent discriminative accuracy (optimism-adjusted C-index: 0.85, 95% CI: 0.83 to 0.87). The average calibration slope (uniform shrinkage factor) was 0.95 (95% CI: 0.82 to 1.08), suggesting little model overfit. The final PI was calculated as 0.95 (uniform shrinkage factor) times the raw PI and the formula for final PI was structured as

1 $$ \mathrm{PI}=-0.002\ast \mathrm{Platelet}-2.399\ast \mathrm{Lymphocyte}+0.044\ast \mathrm{Age}+0.127\ast \mathrm{Neutrophil}+0.468\ast \mathrm{Sex} $$

- Platelet: × 10^9^/L
- Lymphocyte: × 10^9^/L, transformed to lymphocyte ^ 0.5
- Age: in years
- Neutrophil: × 10^9^/L
- Sex: female = 0; male = 1

**Table 2:** Results from multi-variable Fine-Gray model

Variables	Coding	Coefficient	95% CI	***P***
Age	=x^*^	0.046	0.036–0.057	< 0.001
Sex	Dummy (0 = Female, 1 = Male)	0.490	0.179–0.802	0.002
Neutrophil count	=x	0.133	0.109–0.156	< 0.001
Lymphocyte count	Lymphocyte count ^ 0.5	−2.514	−3.192- -1.835	< 0.001
Platelet count	=x	−0.002	−0.004 - -0.001	0.028

^*^x stands for original value

**Fig. 1:**
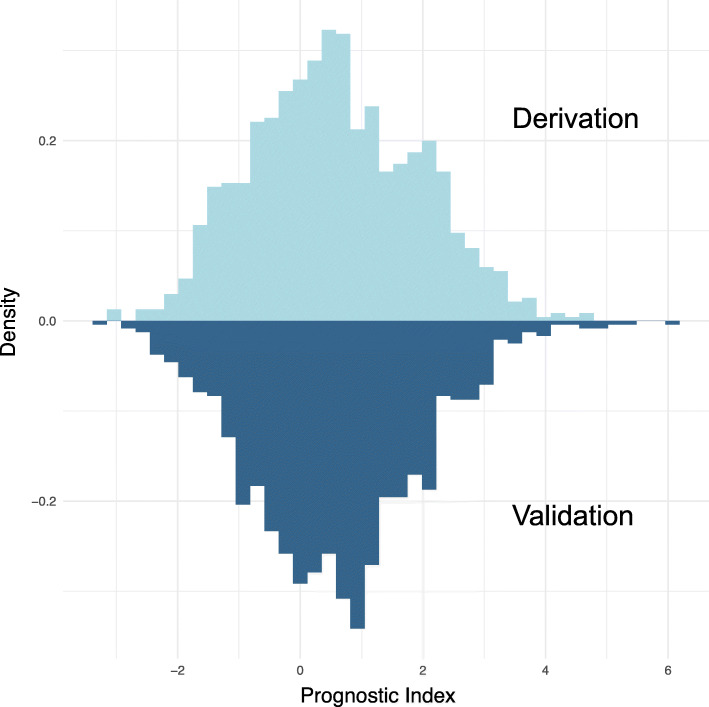
Distribution of the prognostic index of the prognostic model in the derivation and validation cohort; Upper part: derivation cohort; Lower part: validation cohort

**Fig. 2:**
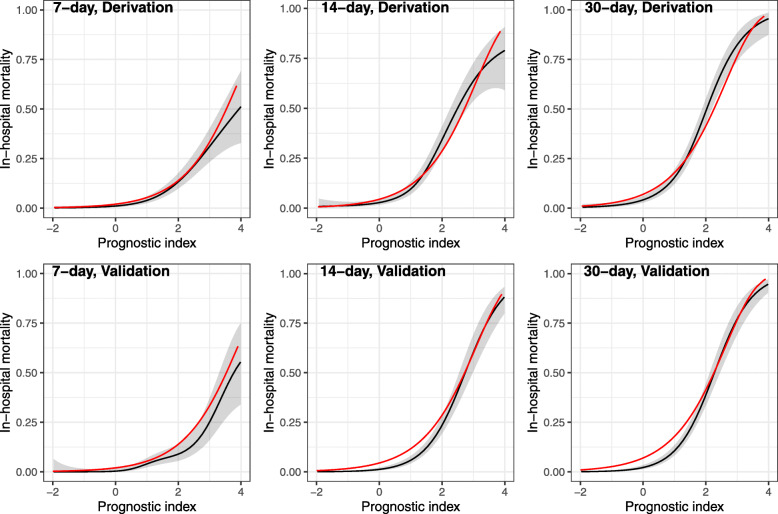
Prediction of 7-day, 14-day and 30-day mortality versus the prognostic index based on the PLANS model (red line); Prediction of 7-day, 14-day and 30-day mortality versus a smooth function of the prognostic index using generalized additive model (black line) together with the 95% confidence band; Upper part: derivation cohort; Lower part: validation cohort

**Fig. 3:**
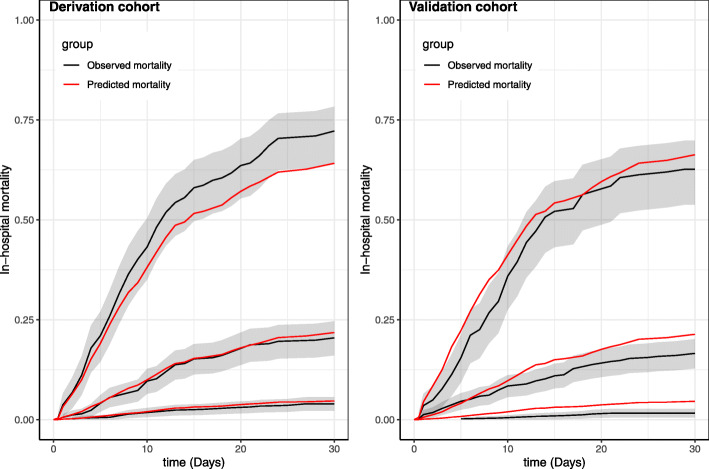
Predicted vs. Observed cumulative incidence curves per risk group in the derivation and validation cohort (Risk groups were defined based on PI. PI range from low risk tp high risk group: ≤ − 0.81, − 0.81 to 0.50, 0.50 to 2.03, and > 2.03. Two lowest group were combined due to the limited death)

The distribution of final PI suggested good discriminative ability of our model (upper panel of Fig. 1). The relationship between PI and 7-day, 14-day and 30-day mortality are presented in Fig. 2. While we observed a slight underestimate of the mortality in the highest risk group, the agreement between predicted mortality curves and the observed mortality curves in the other risk groups suggested good calibration of our model (left panel of Fig. 3). The final formula for the PLANS model and a patient example of how it can be applied in the real clinical practice is depicted in Table 3. Furthermore, an online calculator can be accessed for this calculation: https://plans.shinyapps.io/dynnomapp/.

**Table 3:**
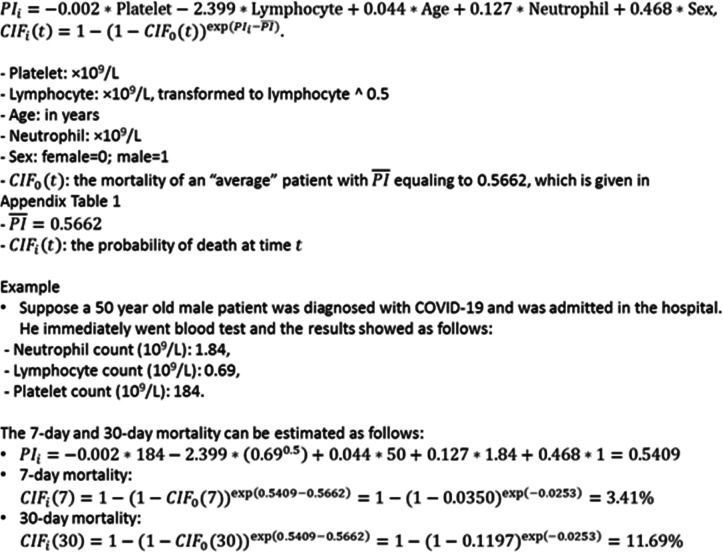
Final prognostic model (PLANS)

### External validation

We applied the PLANS model to the independent cohort of 1031 patients from Tongji Hospital. The distribution of the PIs in the validation cohort was very similar to that in the derivation cohort, suggesting that the excellent discriminative accuracy of our model maintained in the validation cohort (Fig. 1). The resulting C-index showed excellent discriminative accuracy of our model (C-index: 0.87, 95% CI: 0.85 to 0.89). Regarding the calibration accuracy, our model slightly overestimated mortality in each risk group (right panel of Fig. 3). Details about the thresholds and corresponding proportion and death toll included in each risk group are provided (Additional file 1: Appendix Table 2). Jointly considering a close-to-one calibration slope (1.02, 95% CI: 0.92 to 1.12) and good agreement between predicted and observed cumulative incidence curves, our model still suggested good calibration accuracy in the validation cohort.

### Model update

The proposed model may not be directly applied to other areas where the distribution of predictive factors may be different from that in Wuhan. For instance, New York of USA and Lombardy of Italy could have a different distribution of preditor variables compared with Wuhan. Therefore, we used entropy balancing to update proposed model to generalize to their settings [22]. Details about the two updated model, see the Additional file 1: Appendix Text 3.

### Methodology quality assessment

According to the PROBAST, the proposed model was rated as low risk of bias in all four domains: 17 of the total signaling questions were “Yes” and 3 were “Probably Yes”. Rationales of answers were shown in Additional file 1: Appendix Table 6.

## Discussion

We developed a prognostic model (PLANS), using clinical readily available measures of platelet count, lymphocyte count, age, neutrophil count, and sex, to predict in-hospital mortality for COVID-19 patients using two retrospective cohorts in Wuhan, China. This model was first internally validated using bootstrap and then externally validated in an independent cohort in Wuhan. The PLANS model showed excellent discriminative and calibration accuracy.

All the five predictors are routinely collected and some of them have been already well established as the risk factors for in-hospital mortality in previous studies [23]. Recent studies from Italy, the USA, and China [24–26] have also reported that advanced age was a strong predictor of in-hospital mortality as suggested in our study. Compared to previous studies, [27, 28] our study had a more balanced gender composition. Our finding that male gender was associated with increased in-hospital mortality provided further evidence to support the hypothesis of male’s vulnerability to COVID-19 [29, 30]. Our study further confirmed that poor prognosis was associated with higher neutrophil and lower lymphocyte count [31]. On top of that, lymphopenia was found to have a non-linear relation with in-hospital mortality. A meta-analysis of nine studies had reported that thrombocytopenia was significantly associated with the severity of COVID-19 disease, but heterogeneity between studies was high [32]. Given a relatively large sample size and longer follow-up, our study indicated thrombocytopenia was associated with a higher risk of in-hospital mortality. Other studies have shown that several comorbidities (hypertension, diabetes, and coronary heart disease) were associated with poor prognosis [24, 33]. While none of the comorbidities were included in our model, we found that diabetes status would be incorporated when we excluded age from our model. It is plausible as the prevalence of most comorbidities, in particular diabetes, increases with age [25].

Since the outbreak of COVID-19 in Wuhan, a number of prognostic models have been established [7]. A comprehensive systematic review conducted by Wynants and colleagues found that most of these models were of high risk of bias due to several methodological limitations from participant domain to analysis domain [7]. Compared to the previous models, the PLANS model has several strengths. Our derivation cohort had a relatively large sample size with complete information on candidate predictors. While duration of follow up was unclear in most of the previous studies, the patients in our study were followed over a relatively long period, allowing us to perform a time-to-event analysis to predict in-hospital mortality. Furthermore, a competing risk analysis treating discharged alive as a competing event was done in this study to avoid overestimation of mortality. The similar distribution of age and sex in our study to recent large international reports [34, 35] indicates good representativeness of the patient population. External validation of the PLANS model to a large sample of patients showed excellent discrimination and calibration accuracy, indicating the generalizability of the PLANS model in the same city. Furthermore, we explored the possibility of generalizing the PLANS model to New York and Lombardy by using the published summary statistics. Though the adapted models are not recommended being applied before external validation, it might still be a good initiative to develop them and make use of them in the areas where the pandemic is still prevailing. The PLANS model was developed following high methodological standard and rated as low risks of bias in all four domains using PROBAST. Therefore, the PLANS model might be more reliable than most of the published prognostic models in making clinical decisions.

Several limitations should be noted. First, like most of the previous datasets and two main initiatives which created protocols for the investigators, namely, the ‘International Severe Acute Respiratory and emerging Infectious Consortium (ISARIC)’ and the ‘Lean European Open Survey on SARS-CoV-2 Infected Patients (LOESS)’, we only include closed (discharged or dead) COVID-19 cases. However, the resulting bias of unrepresentative sample could be largely offset by the long period of follow-up time. Second, we did have missing data on current smoking status for some patients. Inclusion of smoking status into the current model might improve the model performance. However, a reliable mechanism under the association between smoking and negative progression of COVID-19 is still missing [36]. Third, some potential risk factors confirmed by previous studies, such as D-dimer [31], C-reactive protein [37], lactate dehydrogenase [27, 38], and interleukin-6 [27], were not available in our study. Respiration symptoms were not available either, and inclusion of which might improve the predictive accuracy. However, considering the practicality and validity in clinical application, a simple and interpretable model is usually preferred [39]. In addition, our model showed promising performances with five routinely available predictors, balancing the trade-off between model performance and model practicality.

### Implication for practice

The availability of a prognostic model that can accurately predict in-hospital mortality in COVID-19 patients upon admission to hospital has important implications for practice and policy. The PLANS model may assist physicians to early stratify the patients according to the estimated mortality at 7-day (14-day or 30-day) after admission, thus giving patients targeted supporting care and better allocating the limited medical facilities (e.g. ventilators), especially when critical care capacities are overwhelmed. Several studies showed that physicians have been experiencing guilt when they make clinical decisions that contravene the morals of those making them, e.g. one ventilator, two patients [40, 41]. The PLANS model might be useful to be incorporated into a protocol to assist physicians in making those difficult decisions. Our findings from the model update suggest that our model might be generalized to different countries as well. The model could be validated in the first place and then be used directly if it performs well or after being updated according to local settings [42].

## Conclusions

In summary, the PLANS model can be a guidance model for Chinese hospitals in case of the resurgence of COVID-19. It can also be a useful tool for predicting mortality or triage patients in the countries where COVID-19 is still a pandemic after being validated in their settings. Future studies are warranted about the impact of the PLANS model on clinical practice and decision.

## Supplementary Information

**Additional file 1: **. **Appendix Text 1**. Implementation and estimates of Fine-Gray model. **Appendix Text 2**. Internal validation by bootstrap. **Appendix Text 3**. Two updated models. **Figure 1**. Cumulative mortality for derivation and validation cohort. **Figure 2**. The Schoenfeld residual plots for each predictor, test of proportional hazards. **Table 1**. “Baseline”* mortality (Wuhan, China). **Table 2**. Thresholds and corresponding proportion and death toll included in each risk group. **Table 3.** Basic characteristics used in entropy balancing in Derivation cohort, New York cohort and Lombardy cohort. **Table 4.** “Baseline”* mortality (New York, USA). **Table 5**. “Baseline”* mortality (Lombardy, Italy). **Table 6** Methodology quality assessment based on PROBAST risk of bias assessment tool

**Additional file 2: TRIPOD Checklist**: Prediction Model Development

## Data Availability

The datasets analyzed during the current study are available from the corresponding author on reasonable request.

## Abbreviations

COVID-19: Coronavirus disease 2019
ICU: Intensive care unit
CHD: Coronary heart disease
PI: Prognostic index
CIF: Cumulative incidence function
IQR: Interquartile range
LOS: Length of stay
