# Supplementary material for: Derivation and validation of a prognostic model for predicting in-hospital mortality in patients admitted with COVID-19 in Wuhan, China: the PLANS (platelet lymphocyte age neutrophil sex) model
Source: BMC Infect Dis. 2020 Dec 17;20:959. doi: 10.1186/s12879-020-05688-y (PMC7744735; doi:10.1186/s12879-020-05688-y)
Supplement: Supplementary file 1 — Additional file 1: . Appendix Text 1. Implementation and estimates of Fine-Gray model. Appendix Text 2. Internal validation by bootstrap. Appendix Text 3. Two updated models. Figure 1. Cumulative mortality for derivation and validation cohort. Figure 2. The Schoenfeld residual plots for each predictor, test of proportional hazards. Table 1. “Baseline”* mortality (Wuhan, China). Table 2. Thresholds and corresponding proportion and death toll included in each risk group. Table 3. Basic characteristics used in entropy balancing in Derivation cohort, New York cohort and Lombardy cohort. Table 4. “Baseline”* mortality (New York, USA). Table 5. “Baseline”* mortality (Lombardy, Italy). Table 6 Methodology quality assessment based on PROBAST risk of bias assessment tool [file 12879_2020_5688_MOESM1_ESM.docx]

**Appendix**

TRIPOD Checklist

Text 1. Implementation and estimates of Fine-Gray model

Text 2. Internal validation by bootstrap

Text 3. Two updated models

Figure 1. Cumulative mortality curves for derivation and validation cohort.

Figure 2. The Schoenfeld residual plots for each predictor, test of proportional hazards

Table 1. “Baseline” mortality (Wuhan, China)

Table 2. Thresholds and corresponding proportion and death toll included in each risk group

Table 3. Basic characteristics used in entropy balancing in Derivation cohort, New York cohort and Lombardy cohort

Table 4. “Baseline” mortality (New York, USA)

Table 5. “Baseline” mortality (Lombardy, Italy)

Table 6. Methodology quality assessment based on PROBAST risk of bias assessment tool

**Appendix Text 1. Implementation and estimates of Fine-Gray model**

Since there were no patients lost to follow-up in our study, conventional Cox models gives the same estimate as Fine-Gray models by treating discharged patients as being right censored at maximum follow-up time (defined as 30 days after hospital admission) irrespective of the actual length of hospital stay.(1) A valid nonparametric estimates of the cumulative incidence function could also be obtained by Kaplan-Meier method using this data coding approach.(1)

**Appendix Text 2. Internal validation by bootstrap**

We performed internal validation to estimate the optimism (the level of model overfitting) and adjusted measures of C-index and calibration slope by bootstrapping 1000 samples of the original data. The model derivation process was repeated in each bootstrap dataset and then created 1000 bootstrap models (1000 apparent C-indice). We applied these 1000 bootstrap models to the original dataset (1000 validated C-indice). Optimism in C-index was determined through the difference between the average apparent C-index and the average validated C-index. Optimism-adjusted C-index was obtained by subtracting the optimism from the original C-index. Calibration slope was calculated by estimating the regression coefficient of the PI from the 1000 bootstrap models in the original dataset. Average calibration slope was obtained to be a uniform shrinkage factor.

**Appendix Text 3. Two updated models**

The proposed model may not be directly applied to other areas where the distribution of predictive factors may be different from that in Wuhan. For instance, New York of USA and Lombardy of Italy could have a different distribution of predictor variables compared with Wuhan.(2,3) Therefore, we used entropy balancing to update proposed model to generalize to their settings.(4) First, entropy balancing approach was implemented to estimate a weight that made our derivation data comparable with the New York cohort in terms of the distribution of age, sex, hypertension, CHD, diabetes and malignancy. Second, a weighted Cox regression was used estimate the baseline mortality of the “average” patient in New York by offsetting the PI. Last, the updated prognostic model for New York can be obtained via ${CIF}_{i}\left( t \right)={{1-(1-CIF}_{0}(t))}^{exp({PI}_{i}-\bar{PI})}$, where $\mathrm{CIF}_{0}(t)$ is a New York specific baseline survival, ${PI}_{i}$ is the PI of patient $i$ and $\bar{PI}$ is the PI of the average patient in New York, which was assumed the same as that in Wuhan. Same procedures were implemented to obtain the updated model in Lombardy.

Compared with those in Wuhan, patients in New York and Lombardy were older and prevalent with comorbidities (Appendix Table 3). While Wuhan and New York had similar gender composition, Lombardy had a much higher of proportion of males (Appendix Table 3). With the same PI equaling to 0.566, patients in New York had similar mortality, while those in Lombardy had lower mortality compared with those in our derivation cohort in Wuhan. The final formula for calculating the survival probability for patient $i$ is ${CIF}_{i}\left( t \right)={{1-(1-CIF}_{0}(t))}^{exp({PI}_{i}-\bar{PI})}$, where $\mathrm{CIF}_{0}(t)$ is the mortality of the “average” patient in New York or Lombardy and is given in Appendix Table 4 and Table 5; respectively; ${PI}_{i}$ is the prognostic index of patient $i$ and can be calculated by formula 1; $\bar{PI}$ is the mean value of PIs in the derivation cohort and is 0.5662.

**Reference**

1. Austin PC, Lee DS, D’Agostino RB, Fine JP. Developing points-based risk-scoring systems in the presence of competing risks: Competing Risks and Risk Scores. Statist Med. 2016 Sep 30;35(22):4056–72.

2. Richardson S, Hirsch JS, Narasimhan M, Crawford JM, McGinn T, Davidson KW, et al. Presenting Characteristics, Comorbidities, and Outcomes Among 5700 Patients Hospitalized With COVID-19 in the New York City Area. JAMA [Internet]. 2020 Apr 22 [cited 2020 May 5]; Available from: https://jamanetwork.com/journals/jama/fullarticle/2765184

3. Grasselli G, Zangrillo A, Zanella A, Antonelli M, Cabrini L, Castelli A, et al. Baseline Characteristics and Outcomes of 1591 Patients Infected With SARS-CoV-2 Admitted to ICUs of the Lombardy Region, Italy. JAMA. 2020 Apr 28;323(16):1574.

4. Hainmueller J. Entropy Balancing for Causal Effects: A Multivariate Reweighting Method to Produce Balanced Samples in Observational Studies. Polit anal. 2012;20(1):25–46.


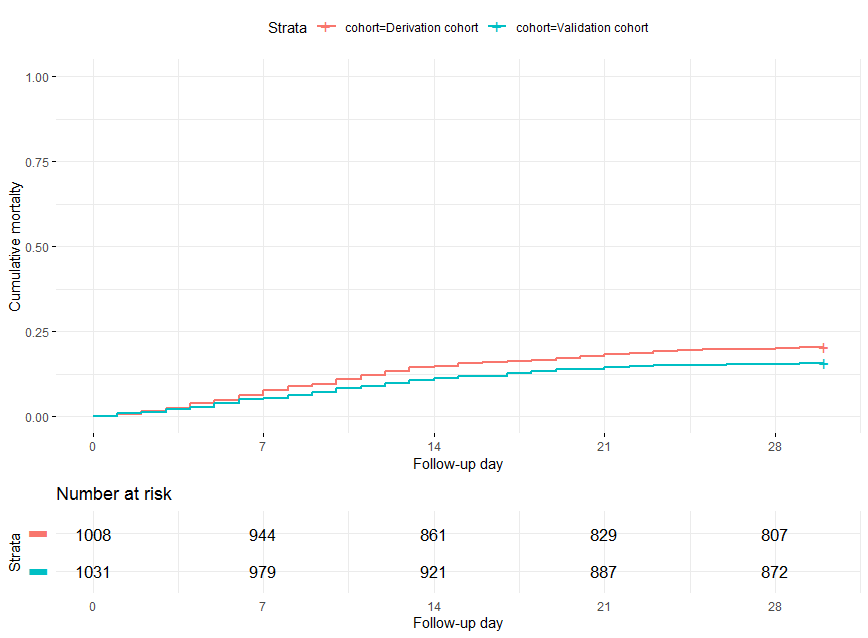


**Figure 1. Cumulative mortality for derivation and validation cohort**


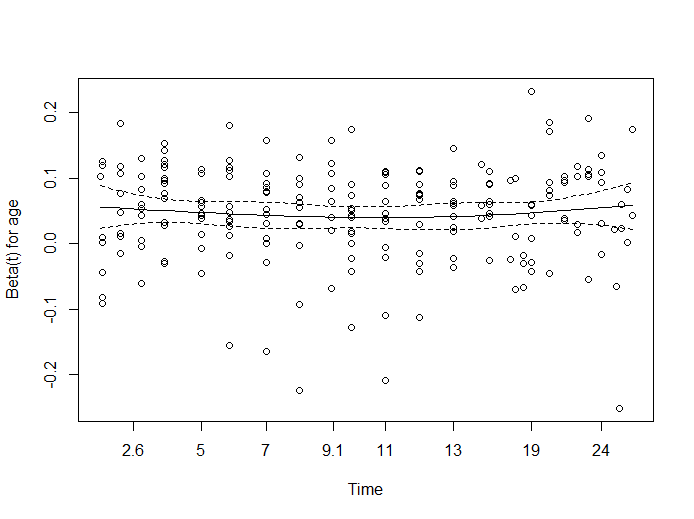

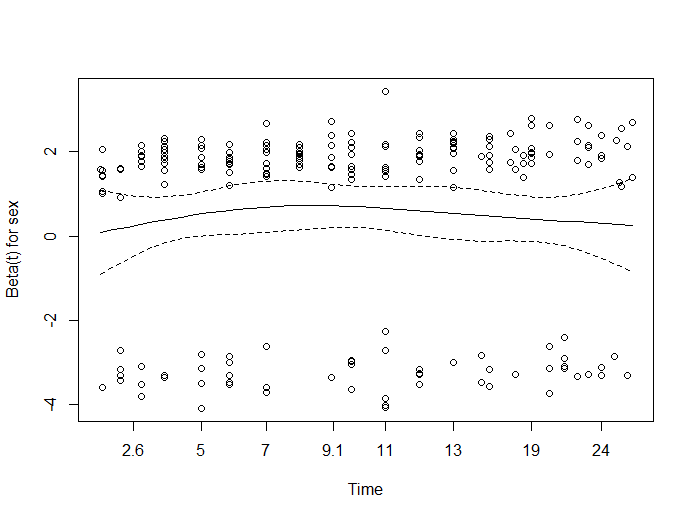

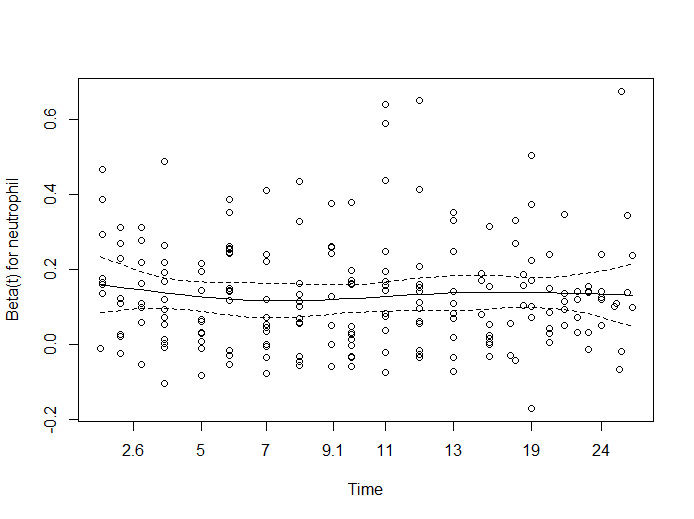

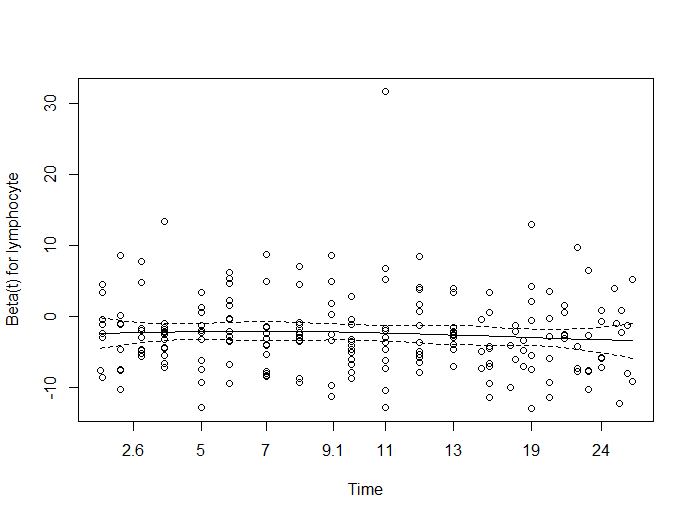

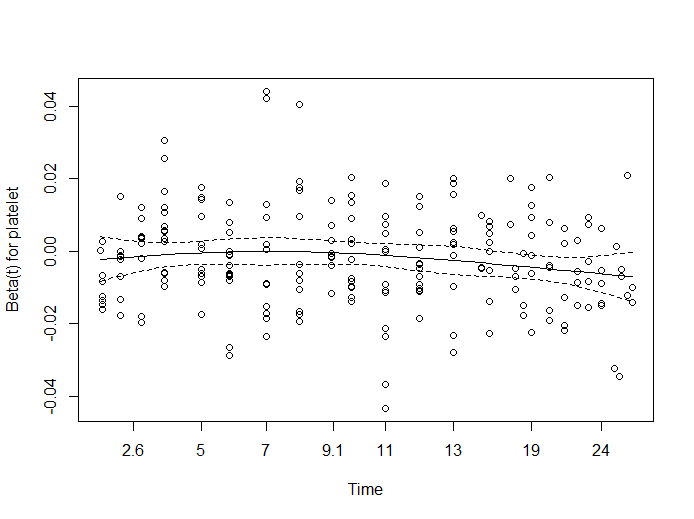


**Figure 2. The Schoenfeld residual plots for each predictor, test of proportional hazards**

**Table 1. “Baseline”* mortality (Wuhan, China)**

| Day | Mortality | Day | Mortality |
| --- | --- | --- | --- |
| 1 | 0.32% | 16 | 8.38% |
| 2 | 0.65% | 17 | 8.58% |
| 3 | 1.04% | 18 | 8.79% |
| 4 | 1.67% | 19 | 9.29% |
| 5 | 2.18% | 20 | 9.74% |
| 6 | 2.85% | 21 | 10.12% |
| 7 | 3.50% | 22 | 10.43% |
| 8 | 4.12% | 23 | 10.82% |
| 9 | 4.55% | 24 | 11.22% |
| 10 | 5.27% | 25 | 11.31% |
| 11 | 6.02% | 26 | 11.39% |
| 12 | 6.81% | 27 | 11.47% |
| 13 | 7.51% | 28 | 11.63% |
| 14 | 7.71% | 29 | 11.80% |
| 15 | 8.24% | 30 | 11.97% |

* The baseline refers to an “average” patient with PI 0.5662.

**Table 2. Thresholds and corresponding proportion and death toll included in each risk group**

| Risk group | Range in PI | Derivation | | Validation | |
| --- | --- | --- | --- | --- | --- |
|  |  | Proportion | Death | Proportion | Death |
| 1. Low risk | <= -0.81 | 16.0% | 3 | 15.6% | 0 |
| 2. Low-Intermediate risk | -0.81 to 0.50 | 34.0% | 17 | 32.5% | 8 |
| 3. Moderate risk | 0.50 to 2.03 | 34.0% | 70 | 38.6% | 65 |
| 4. High risk | > 2.03 | 16.0% | 117 | 13.8% | 89 |

**Table 3. Basic characteristics used in entropy balancing in Derivation cohort, New York cohort and Lombardy cohort**

|  | Derivation cohort  (n=1008) | New York cohort  (n=5700) |  | Lombardy cohort (n=1591) |
| --- | --- | --- | --- | --- |
| Age, years | 55 (44-65) | 63 (52-70) |  | 63 (56-70) |
| Sex, female | 439 (43.6%) | 2263 (39.7%) |  | 287 (18%) |
| Hypertension | 232 (23.0%) | 3026 (56.6%) |  | 509 (49%) |
| Coronary heart disease | 32 (3.2%) | 595 (11.1%) |  | 223 (21%) |
| Diabetes | 110 (10.9%) | 1808 (33.8%) |  | 180 (17%) |
| Malignancy | 31 (3.1%) | 320 (6%) |  | 81 (8%) |

**Table 4. “Baseline”* mortality (New York, USA)**

| Day | Mortality | Day | Survival |
| --- | --- | --- | --- |
| 1 | 0.33% | 16 | 8.11% |
| 2 | 0.95% | 17 | 8.19% |
| 3 | 1.33% | 18 | 8.34% |
| 4 | 2.04% | 19 | 9.02% |
| 5 | 2.42% | 20 | 9.48% |
| 6 | 3.31% | 21 | 9.94% |
| 7 | 4.01% | 22 | 10.45% |
| 8 | 4.43% | 23 | 10.68% |
| 9 | 4.82% | 24 | 11.20% |
| 10 | 5.40% | 25 | 11.24% |
| 11 | 6.04% | 26 | 11.26% |
| 12 | 6.52% | 27 | 11.29% |
| 13 | 7.19% | 28 | 11.36% |
| 14 | 7.36% | 29 | 11.72% |
| 15 | 8.01% | 30 | 11.94% |

* The baseline refers to an “average” patient with PI 0.5662.

**Table 5. “Baseline”* mortality (Lombardy, Italy)**

| Day | Mortality | Day | Mortality |
| --- | --- | --- | --- |
| 1 | 0.36% | 16 | 7.48% |
| 2 | 0.80% | 17 | 7.59% |
| 3 | 1.18% | 18 | 7.72% |
| 4 | 1.72% | 19 | 8.34% |
| 5 | 2.16% | 20 | 8.60% |
| 6 | 2.77% | 21 | 8.72% |
| 7 | 3.25% | 22 | 9.51% |
| 8 | 3.73% | 23 | 9.82% |
| 9 | 4.02% | 24 | 10.11% |
| 10 | 4.59% | 25 | 10.13% |
| 11 | 5.45% | 26 | 10.16% |
| 12 | 5.94% | 27 | 10.21% |
| 13 | 6.39% | 28 | 10.32% |
| 14 | 6.82% | 29 | 10.44% |
| 15 | 7.36% | 30 | 10.72% |

* The baseline refers to an “average” patient with PI 0.5662.

**Table 6 Methodology quality assessment based on PROBAST risk of bias assessment tool**

| **Question** | **Answer** | **Rationale** |
| --- | --- | --- |
| **Domain 1: Participants** |  |  |
| 1·1 Were appropriate data sources used, e.g., cohort, RCT or nested case-control study data? | Yes | Cohort design was used. |
| 1·2 Were all inclusions and exclusions of participants appropriate? | Probably yes | The patients had no event (either death or discharge) at the end of follow-up date were excluded from the model development cohort. This is not model developers’ choice, but due to data availability. Given the time between end of accrual and end of follow-up was 40 days, the actual proportion of excluded patients was very small, and the potential risk of bias can be mitigated. |
| **Overall risk of bias of Domain 1** | **Low risk of bias** |  |
| **Domain 2: Predictors** |  |  |
| 2·1 Were predictors defined and assessed in a similar way for all participants? | Probably yes | Most of the predictors were patient characteristics and laboratory tests, which had standard definition. The scope of chronic disease history may be slightly different between the two hospitals. In general, all predictors can be considered as assessed in a similar way. |
| 2·2 Were predictor assessments made without knowledge of outcome data? | Yes | All predictors were collected at patients admission. |
| 2·3 Are all predictors available at the time the model is intended to be used? | Yes | All predictors included in the final model were patient characteristics and laboratory tests, which were easy to access· |
| **Overall risk of bias of Domain 2** | **Low risk of bias** |  |
| **Domain 3: Outcome** |  |  |
| 3·1 Was the outcome determined appropriately? | Yes | In-hospital death was an event of interest for admitted patients with COVID-19. Discharge was properly considered as a competing risk event of in-hospital death, this is an advantage of this study compared with other studies (in which discharge was censored). |
| 3·2 Was a pre-specified or standard outcome definition used? | Yes | In-hospital death was a hard end-point. |
| 3·3 Were predictors excluded from the outcome definition? | Yes | In-hospital death was objective. |
| 3·4 Was the outcome defined and determined in a similar way for all participants? | Yes | In-hospital death was objective. |
| 3·5 Was the outcome determined without knowledge of predictor information? | Yes | In-hospital death was objective. |
| 3·6 Was the time interval between predictor assessment and outcome determination appropriate? | Yes | All patients were staying in the hospitals and continuously followed up till discharge, death or the end of follow-up (which was 40 days after end of accrual). The time interval between predictor assessment and outcome determination is sufficient. |
| **Overall risk of bias of Domain 3** | **Low risk of bias** |  |
| **Domain 4: Analysis** |  |  |
| 4·1 Were there a reasonable number of participants with the outcome? | Yes | The total number of candidate variables was 11, and the number of events was 211, so the number of events per variable (EPV) =211/11≈19, which was reasonable. |
| 4·2 Were continuous and categorical predictors handled appropriately? | Yes | Functional form of the relation between continuous variables and outcome was investigated, and suitable variable transformations were performed in case the linear assumption did not hold. No categorization was done for continuous variables. |
| 4·3 Were all enrolled participants included in the analysis? | Yes | All patients met the inclusion criteria were included in the analysis. |
| 4·4 Were participants with missing data handled appropriately? | Probably yes | Predictors with high percentage of missing value were excluded from the final model. |
| 4·5 Was selection of predictors based on univariable analysis avoided? | Yes | Variable selection was based on multivariable analysis, and univariable was performed for investigating the non-linear relation. |
| 4·6 Were complexities in the data (e.g., censoring, competing risks, sampling of controls) accounted for appropriately? | Yes | Discharge was properly considered as a competing risk event of in-hospital death in the analysis. |
| 4·7 Were relevant model performance measures evaluated appropriately? | Yes | Discrimination was assessed by C-statistics, and calibration curve was used to assess calibration. |
| 4·8 Were model overfitting and optimism in model performance accounted for? | Yes | Optimism in model performance was adjusted with bootstrapping internal validation, a shrinkage factor was multiplied to the linear predictor to prevent overfitting. |
| 4·9 Do predictors and their assigned weights in the final model correspond to the results from multivariable analysis? | Yes | Coefficients of the multivariable Cox model was directly used in the calculation of the prognostic index (PI). |
| **Overall risk of bias of Domain 4** | **Low risk of bias** |  |
